# No apparent role for the Wari insulator in transcriptional regulation of the endogenous *white* gene of *Drosophila melanogaster*

**DOI:** 10.17912/micropub.biology.000702

**Published:** 2023-04-05

**Authors:** Gordian Born, Dimi Bieli, Mario Metzler, Daryl M Gohl, Markus Affolter, Martin Müller

**Affiliations:** 1 Biozentrum der Universität Basel, Basel, Switzerland; 2 Arcondis, Reinach, Switzerland; 3 Mabylon AG, Schlieren, Switzerland; 4 Oliver Wyman AG, Zürich, Switzerland; 5 University of Minnesota Genomics Center, Minneapolis, MN, USA

## Abstract

Chromatin insulators have been proposed to play an important role in chromosome organization and local regulatory interactions. In *Drosophila* , one of these insulators is known as Wari. It is located immediately downstream of the 3’ end of the *white* transcription unit. Wari has been proposed to interact with the *white* promoter region, thereby facilitating recycling of the RNA polymerase machinery. We have tested this model by deleting the Wari insulator at the endogenous *white* locus and could not detect a significant effect on eye pigmentation.

**Figure 1. Genetic engineering of new white alleles and their effect on eye pigmentation:**
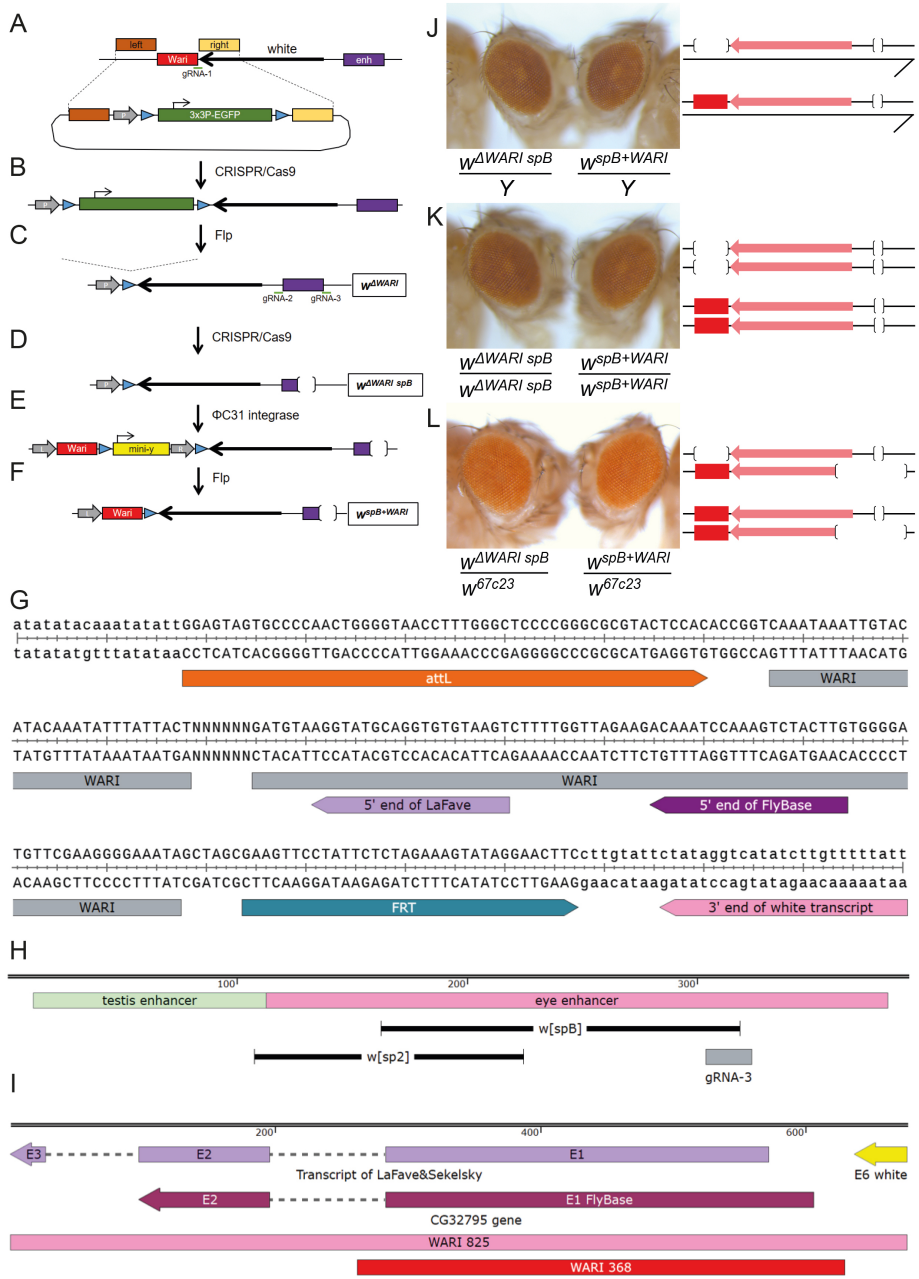
**(A)** Generation of *w ^ΔWari-GFP^* : The *white* gene (black arrow), Wari (red box) and the *white* enhancer (purple box) are shown. The position of gRNA-1 is indicated by a green line to the right of Wari. In addition, the 500 bp feet required for homologous recombination are shown on either side of Wari (brown and yellow boxes). Below, the relevant genetic elements present on donor plasmid pMM085 are depicted: grey arrow represents an attP site, blue arrowheads are FRT sites, green box corresponds to 3x3P-EGFP marker. Note that genetic elements in 1A-F are not drawn to scale. **(B)** Generation of *w ^ΔWari-GFP^* by knock-in of 3x3P-EGFP marker in place of Wari. **(C)** Generation of *w ^ΔWari^* by Flp-induced deletion of 3x3P-EGFP marker. **(D)** Generation of *w ^ΔWari-spB^* by CRISPR/Cas9-mediated deletion of eye enhancer. The gRNAs used for this purpose are indicated in Figure 1C (green lines below purple enhancer element labeled with gRNA-2 and gRNA-3). **(E)** Generation of *w ^spB+Wari-yellow^* by integrase-mediated insertion of reentry plasmid pDB154. Grey arrows indicate attL and attR sites. Yellow box depicts the *mini-yellow* marker. **(F)** Generation of *w ^spB+Wari^* by Flp-mediated deletion of the *mini-yellow* marker. The names of the three alleles described in the text are indicated in (C) *w ^ΔWARI^* , (D) *w ^ΔWari spB^* and (F) *w ^spB+Wari^* . **(G)** In *w ^spB+Wari^* , the 364 bp Wari element (in grey) is flanked by an attL (in orange) and an FRT site (in blue). The latter is located 8 bp downstream of the 3’ end of the single *white* transcript w-RA (indicated in pink; sequence information on the *white* transcript taken from FlyBase genome release R6.47). Note that 249 bp of Wari are missing at the NNNNNN position. The small arrows in light and dark magenta indicate the 5’ end of CG32795 transcripts of LaFave and Sekelsky (2011) and FlyBase, respectively. **(H)** The location of gRNA target site gRNA-3 (in gray), the *white* testis and eye enhancers (in green and pink, respectively; indicated according to Qian et al. 1992) and of the two deletions *w ^sp2^* and *w ^spB^* (black lines indicate the region missing in these deletions) are shown. *w ^spB^* initiates in the gRNA-3 target site and extends distally where it overlaps by 62 bp with *w ^sp2^* . gRNA-2 target site not indicated because it is located distally to testis enhancer. Flanking DNA of *w ^sp2^* : AACGTCAATG and TGTGTGTTTG **.** Flanking DNA of *w ^spB^* : CTTATGGGGC and AAAAATGGCA. **(I)** The 5’ end of gene *CG32795* is located immediately downstream to the 3’ end of the *white* gene. At the top is a scale bar for the 676 bp genomic fragment shown in this panel. Below, the 3’ end of *white* (in yellow; E(xon)6 of *white* transcript) is shown. To its left, the 5’ end of a transcript is depicted in light purple. It was detected in several independently isolated hypomorphic P-element alleles (LaFave and Sekelsky 2011). Below, the first two exons of gene *CG32795* (in dark purple) are shown. This transcript corresponds to isoform CG32795-RD (sequence information from genome release R6.47). At the bottom, the distal part of Wari 825 (in pink) and Wari 368 (in red) are shown. Wari 825 was the starting point for the dissection of the insulator activity detected downstream of *mini-white* , which resulted in the definition of Wari 368 (Chetverina et al. 2008). The most distal 46 bp of Wari 825 are part of pCaSpeR-2’s adjacent P-element end and thus not indicated. Consequently, the distal end of Wari 825 corresponds to that of the LaFave transcript. Note that the transcription start sites of the FlyBase and the LaFave transcripts are contained in Wari 368. Transcription start sites of other CG32795 isoforms (not indicated) are further downstream and not included in Wari 825 nor in Wari 368. ( **J)** Males with genotypes *w ^ΔWari spB^ /Y* and *w ^spB+Wari^ /Y* are shown. **(K)** Females with genotypes *w ^ΔWARI spB^ /w ^ΔWari spB^* and *w ^spB+Wari^ /w ^spB+Wari^* are shown. **(L)** Females with genotypes *w ^ΔWari spB^ /w ^67c23^* and *w ^spB+Wari^ /w ^67c23^* are shown. To the right of each picture, diagrams of the two genotypes are depicted (upper diagram left fly, bottom diagram right fly). Note that compared to *Oregon-R* , eye pigmentation is considerable reduced in *w ^ΔWari spB^* males and females, indicating that the *w ^spB^* enhancer deletion is largely disabling the eye enhancer. However, dosage compensation is still taking place since homozygous females have clearly darker eyes than heterozygous sisters (compare eye colors in Figure 1K and 1L). In none of the three genotypes, the presence of the Wari element leads to a significant increase in eye pigmentation. Approximate location of *Df(1)w ^67c23^* break points at 5’ end of *white* indicated according to Moschetti et al. (2004).

## Description

The *Drosophila white* ( *w* ) gene has been at the heart of several important discoveries in genetics and molecular biology (reviewed in Hazelrigg 1987; Green 2010; Mohr 2018). It is an X-linked gene with a rather simple structure. A single transcript is expressed under the control of a few tissue-specific enhancers. For example, a 270 bp eye enhancer is located about 1200 bp upstream of the *white* transcription start site. This enhancer is essential for efficient expression of *white* in the eye where it plays an essential role in pigment deposition (Davison et al. 1985; Qian et al. 1992) . While wild-type flies have red eyes, *white* null alleles have white eyes. Hypomorphic *white* mutants can have a continuum of allele-specific intermediate eye colors ranging from pale yellow to almost wild-type red. These differences in eye pigmentation are very useful for scoring phenotypes in a semi-quantitative manner. The easy-to-score white phenotype was employed for the construction of numerous vectors used for the generation of transgenic flies. Such vectors contain the so-called *mini-white* gene, a derivative of the endogenous *white* gene from which a large part of the first intron has been deleted and which lacks all known tissue-specific enhancers (Pirrotta 1988) .

Thanks to its simple organization and its obvious phenotype, *mini-white* in combination with its eye enhancer became the workhorse of researchers interested in chromatin insulators (Kellum and Schedl 1991, 1992; reviewed in Geyer 1997) . Insulators were proposed to subdivide chromatin into separate looped domains by pairwise association of two insulators. Furthermore, it was proposed that regulatory elements located in separate chromatin loops are unable to interact with each other. Therefore, putative insulators were tested by insertion in between the *mini-white* gene and the *white* eye enhancer. Transgenic flies were generated and the eye color phenotype was scored. The less pigmented the eye appeared, the stronger the effect of the insulator was ranked.

When more sophisticated arrangements of insulators and reporter genes were analyzed, Chetverina et al (2008) found that the *mini-white* reporter itself was associated with an insulator-like element. It is confined to a 368 bp fragment that is located only 8 bp distal to the 3’ end of the *white* gene (see Figure 1I ) and is called Wari (acronym for w hite a butting r esident i nsulator). Later studies reported that in S2 cells, Wari was bound by insulator proteins such as CP190. In addition, in transgene inserts, Wari was found to functionally interact with the *white* promoter (Erokhin et al. 2010, 2011) . These authors proposed that insulators like Wari that are located downstream of a gene could support a loop that brings together a promoter and a transcriptional terminator. They further suggested that such loop formation might be a common feature of gene activation that serves to promote efficient transcriptional elongation and reinitiation by facilitating RNAP II recycling from the terminator to the promoter.

A simple prediction from this model is a noticeable effect on eye pigmentation caused by deletion of Wari at the endogenous *white* locus. To test such a scenario, we deleted Wari by CRISPR/Cas9-mediated homologous recombination in *Oregon-R* flies (for details on the generation of all alleles presented in this study, see Figure 1A -F and Materials). The eye color of *w ^ΔWari^* flies was indistinguishable from that observed in *Oregon-R* controls. It is known that the eye color of homozygous *w ^+^* and heterozygous *w ^+^ /w ^-^* females is essentially identical. Thus, we reasoned that in *w ^ΔWari^* flies, a phenotypic effect could be masked by the recessive nature of the *white* locus. Therefore, we sensitized the *white* locus by introducing a 156 bp deletion in the *white* eye enhancer in the context of the *w ^ΔWari^* allele. The new allele is called *w ^ΔWari-spB^* ( *spB* stands for “ *spotted-Basel* ”; see Figure 1H ). As expected, compared to *w ^ΔWari^* , the eye color of these flies was significantly reduced (see Figure 1J -L). This observation is of utmost importance for the interpretation of our experiment. With the introduction of *w ^spB^* , the eye color of *w ^ΔWari-spB^* has become dosage-dependent; eye pigmentation of homozygous and heterozygous *w ^ΔWari-spB^* females were easily distinguishable (compare eye colors in Figures 1K and 1L). This strongly suggests that upon introduction of Wari into *w ^ΔWari-spB^* , even a moderate increase in eye pigmentation should become detectable if Wari indeed contributes to *white* transcriptional activity. Towards that end, the attP landing site on *w ^ΔWari-spB^* was used to re-introduce Wari at the 3’ end of the *white* gene. This allele is referred to as *w ^spB+Wari^* . Importantly, we observed no significant difference between the eye color of *w ^ΔWari-spB^* and *w ^spB+Wari^* flies, suggesting that Wari does not play a significant role in *white* gene transcription (see Figure 1J -L).

A vast body of literature on insulator function has accumulated over the last two decades (reviewed in Kyrchanova and Georgiev 2013). Apart from detailed studies on insulator elements in the bithorax complex (reviewed in Maeda and Karch 2015), essentially all these studies relied on the analysis of transgenic constructs. The data suggests that insulators are not necessarily organizers of higher order chromatin structure but rather facilitators of local enhancer-promoter interactions. We have now tested this model for the Wari insulator at its endogenous genomic location. Our observations suggest that the proposed loop-formation between Wari and the *white* promoter does not play a significant role in *white* gene transcription in the eye. This raises the question why in transgenic eye color assays, Wari can act as an enhancer blocker or interact with other insulators. Based on the most recent fly genome release R6.47, the 5’ end of gene CG32795 is located immediately distal to the 3’ end of *white* (see Figure 1I ). In the context of our assay system (see Figure 1G ), FlyBase-annotated transcript CG32795-RD starts 23 bp distal of the FRT, the transcript of LaFave 57 bp distal to it (LaFave and Sekelsky 2011) . *Drosophila* promoters are in general less than 50 bp long (reviewed in Vo Ngoc et al. 2019). Hence, there should be space enough to accommodate the RNAP II machinery as well as insulator binding proteins that are frequently also bound to promoters. It is therefore conceivable that the presence of such proteins on Wari are responsible for its insulator-like behavior on transgenes. However, when analyzed at the endogenous locus, it appears as Wari has no obvious role in *white* gene regulation.

## Methods

Generation of new white alleles

*w ^ΔWari-GFP^* : For CRISPR/Cas9, the following three plasmids were injected into *Oregon-R* embryos: 1. Donor plasmid pMM085 (see Figure 1A ; synthesized by Genewiz). It consists of (1) 500 bp left foot, (2) 55 bp attP, (3) 34 bp FRT, (4) 1262 bp 3x3P-EGFP as selectable marker, (5) 34 bp FRT and (6) 500bp right foot. 2. gRNA-1 plasmid pDB149 (“gRNA-1” in Figure 1A ; gRNA sequence is: 5’-GGGAAATACTTGTATTCTAT-3’). 3. Cas9 helper plasmid pBS-Hsp70-Cas9 (Gratz et al. 2014) . In a successful CRISPR/Cas9 event, homologous recombination leads to the deletion of a 364 bp Wari fragment and the integration of an attP-FRT-3x3P-EGFP-FRT cassette (see Figure 1B ). Following injection in *Oregon-R* embryos, injectees were grown to adulthood. 30 G0 crosses were set up, of which 18 were fertile. One G0 male produced many GFP ^+^ larvae, indicating a high probability for the success of the planed homologous recombination. GFP ^+^ larvae were transferred into a fresh vial and grown to adulthood. As expected for an X-linked genome editing event in the male germline, all adult flies emerging from this vial were females. They were used to establish an FM7c balanced stock. PCR and sequencing confirmed successful genome editing according to the design on donor plasmid pMM085. The new *white* allele is referred to as *w ^ΔWari-GFP^* . Like all alleles described in this work, it is homozygous viable and maintained as a homozygous stock.

*w ^ΔWari^* : the 3x3P-FGFP marker was deleted by Flp treatment.

*w ^ΔWari-spB^* : a deletion of the *white* enhancer was attempted with two gRNAs flanking the eye enhancer of the *white* gene (gRNA-2 and gRNA-3 in Figure 1C ). They are: gRNA-2 (on plasmid pDB151; gRNA sequence is: 5’-ATTTGCTGACGACGATTAAG-3’) and gRNA-3 (on plasmid pDB152; gRNA sequence is: 5’- GCAGCGAAAGAGCTGAAAAA-3’). These two plasmids were injected together with the Cas9 helper plasmid pBS-Hsp70-Cas9 into *w ^ΔWari^* embryos. From the surviving injectees, 14 fertile crosses were obtained. The progeny of these were screened for flies with the typical *w ^sp^* eye color phenotype (Davison et al. 1985) . Two such candidates could be isolated from 1/14 crosses and FM7c balanced stocks were established. In addition to the deletion at the 3’ end of *white* , PCR and sequencing proved the existence of an identical 156 bp deletion in both candidates. This deletion removes about 60% of *white* eye enhancer. It overlaps by 62 bp with the 117 bp *w ^sp2^* deletion (see Figure 1H ). The new allele is referred to as *w ^ΔWari-spB^* . In preparation for the next step, a *y ^1^ w ^ΔWari-spB^* recombinant was generated by meiotic recombination.

*w ^spB+Wari-yellow^* : the attP landing site present on the *y ^1^ w ^ΔWari-spB^* chromosome was used to reintroduce the 364 bp Wari element normally present at the 3’ end of the *white* gene. Compared to the Wari 368 of Chetverina et al. (2008), the most distal four bases are missing (GGAG). The reentry plasmid pDB154 was injected together with the ΦC31 integrase helper plasmid pBS130 into *y ^1^ w ^ΔWari-spB^* embryos. The reentry plasmid pDB154 was constructed in the following way. First, a cassette consisting of attP-Wari-FRT followed by unique KpnI and SalI sites was synthesized by Genewiz. This plasmid is called pDB153 and lacks a selectable marker. Therefore, pDB153 was cut with KpnI and SalI and ligated with a *mini-yellow* KpnI/SalI-fragment isolated from piBLLFY. The resulting plasmid is pDB154 and was used to generate a Wari insert in landing site *w ^ΔWari-spB^* . Unfortunately, the *mini-yellow* marker on pDB154 was not detected after surviving G0 flies were crossed with *y w* partners. Whether this marker remains silent because of a position effect or whether it contains an unknown lesion in the *mini-yellow* gene has not been further analyzed. Rather, 43 F1 flies were selected at random and crossed back with *y w* partners. After a few days, the 43 candidates were sacrificed for PCR. One of the 43 candidates was identified as the desired transgenic. It is called *w ^spB+Wari-yellow^* .

*w ^spB+Wari^* : in a last step, the *mini-yellow* marker present in *w ^spB+Wari-yellow^***** flies was removed by Flp treatment. This allele is called *w ^spB+Wari^* . The sequence details in the Wari and *white* enhancer regions of this stock are depicted in Figure 1G and 1H.

Scoring of eye pigmentation

Necessary crosses were reared on standard cornmeal food and kept at 25 degrees Celsius. In the next generation, flies were collected in a four-hour window and aged for 7 days before eye pigmentation was scored. Pictures were taken with a Leica M125 binocular equipped with a Leica flexacam C3 camera.

Deficiency *w ^67c23^* corresponds to the one contained in Bloomington stock 6599 ( *y ^1^* *w ^67c23^* ).
